# Unconventional Topological Weyl Dipole Phonon

**DOI:** 10.1002/advs.202504812

**Published:** 2025-06-17

**Authors:** Jianhua Wang, Yang Wang, Feng Zhou, Wenhong Wang, Zhenxiang Cheng, Shifeng Qian, Xiaotian Wang, Zhi‐Ming Yu

**Affiliations:** ^1^ Institute of Quantum Materials and Devices Tiangong University Tianjin 300387 China; ^2^ Institute for Superconducting and Electronic Materials Faculty of Engineering and Information Sciences University of Wollongong Wollongong 2500 Australia; ^3^ Key Lab of Advanced Optoelectronic Quantum Architecture and Measurement (MOE) Beijing Key Lab of Nanophotonics & Ultrafine Optoelectronic Systems and School of Physics Beijing Institute of Technology Beijing 100081 China; ^4^ Anhui Province Key Laboratory for Control and Applications of Optoelectronic Information Materials Department of Physics Anhui Normal University Wuhu Anhui 241000 China

**Keywords:** higher‐order topology, hinge states, topological phonons, Weyl phonons

## Abstract

A pair of Weyl points (WPs) with opposite topological charges can exhibit an additional higher‐order *Z*_2_ topological charge, giving rise to the formation of a *Z*_2_ Weyl dipole (WD). Owing to the nontrivial topological charge, *Z*_2_ WDs should also appear in pairs, and the WPs within each *Z*_2_ WD can not be annihilated when meeting together. As a novel topological state, the topological Weyl dipole (TWD) phase has garnered significant attention, yet its realization in crystalline materials remains a challenge. Here, through first‐principles calculations and theoretical analysis, the existence of the nontrivial unconventional WD phase is demonstrated in the phonon spectra of the *P*6_3_ type Y(OH)_3_. Particularly, the nontrivial unconventional WD in this system is protected by a quantized quadrupole moment, and it is distinguished from conventional WD, as it comprises an unconventional charge‐3 WP with charge of –3 and three conventional charge‐1 WPs with charge of +1. Consequently, the nontrivial unconventional WD phase in Y(OH)_3_ features unique 2D sextuple‐helicoid Fermi‐arc states on the top and bottom surfaces, protected by the topological charges, as well as 1D hinge states that connect the two nontrivial unconventional WDs along the side hinges, guaranteed by the quantized quadrupole moment.

## Graphical abstract

A new higher‐order topological phonon state is proposed, which integrates an unconventional Weyl dipole phonon (a charge‐3 Weyl point and three charge‐1 Weyl points) with higher‐order topology. Moreover, the two Weyl dipoles in Y(OH)_3_ exhibit the sextuple‐helicoid surface and hinge boundary states from the nonzero topological charge (±6) and the nontrivial quadrupole moment (*Q*_*WD*_ = 1/2), respectively.

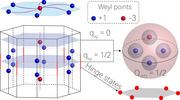

## 1 Introduction

**Figure 1:**
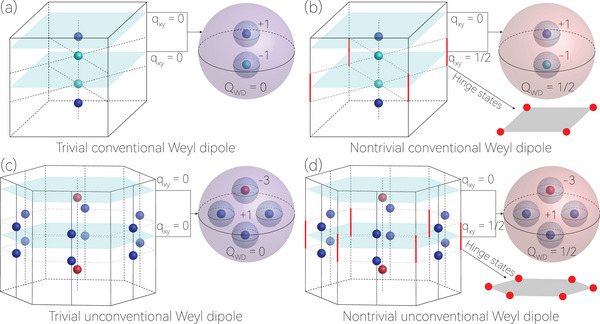
Panels (a) and (b) are schematic illustrations of trivial and nontrivial conventional WDs. The conventional WD consists of two WPs with opposite topological charges ±1. Panels (c) and (d) are schematic illustrations of trivial and nontrivial unconventional WDs. The unconventional WD consists of one charge‐3 WP with topological charge of −3 and three charge‐1 WPs with topological charge of +1. The nontrivial WDs shown in (b) and (d) are characterized by a quantized quadrupole moment *Q*_*WD*_ = 1/2.

The investigation into topological quantum states enhances our understanding of electronic behavior in solid materials.^[^ ^1^, ^2^, ^3^, ^4^, ^5^ ^]^ Thus far, various topological band crossings have been proposed,^[^ ^5^ ^]^ including nodal points^[^ ^6^, ^7^, ^8^, ^9^, ^10^, ^11^, ^12^, ^13^, ^14^ ^]^ and high‐dimensional geometric shapes.^[^ ^15^, ^16^, ^17^, ^18^, ^19^, ^20^ ^]^ Notably, the WPs, exhibiting 0D twofold degeneracy in 3D momentum space and carrying topological *Z* charge defined by the topological charge, have been regarded as one of the most important topological degeneracies. WPs usually appear in pairs with opposite topological charges. The schematic of the classic Weyl pair, composed of two conventional WPs characterized by topological charges of ±1, is shown in **Figure** **1**a. The unconventional WD contains unconventional WP with topological charge‐n (n = 2, 3, 4), such as one charge‐3 WP and three charge‐1 WPs, as shown in Figure 1c. Due to the bulk‐boundary correspondence, topological Weyl phases should have a topologically protected 2D Fermi‐arc state on the surface of the system.

The recent discovery of higher‐order topological phases,^[^ ^21^, ^22^, ^23^, ^24^, ^25^, ^26^, ^27^, ^28^, ^29^, ^30^, ^31^, ^32^, ^33^, ^34^, ^35^, ^36^, ^37^, ^38^, ^39^, ^40^, ^41^, ^42^, ^43^, ^44^, ^45^ ^]^ which host boundary states in at least two dimensions lower than the 3D bulk (such as 1D hinge state or 0D corner state), has unveiled a novel unconventional bulk‐boundary correspondence that extends beyond the conventional one. Recently, higher‐order topological Weyl semimetals have been proposed,^[^ ^27^, ^28^, ^30^ ^]^ where the conventional and second‐order WPs are respectively defined as critical points that separate quantum anomalous Hall insulator (QAHI) and normal insulator and that separate QAHI and a higher‐order quadrupole topological insulator (HOQTI) in momentum space.^[^ ^28^, ^46^ ^]^ This definition further implies that a combination of conventional and second‐order WPs can give rise to a nontrivial conventional WD,^[^ ^28^, ^47^ ^]^ which separates a normal insulator and a HOQTI. Such a nontrivial WD is characterized by a second‐order *Z*_2_ topological charge, namely, a quantized quadrupole moment *Q*_*WD*_, as illustrated in Figure 1b. Therefore, the *Z*_2_ WD should also come in pairs, similar to the WPs and the *Z*_2_ nodal lines. Moreover, the *Z*_2_ topological charge guarantees the appearance of second‐order boundary states along the hinge of the TWD phases, as depicted in Figure 1b,d. In ref. [48], the WD protected by a staggered topological charge is also proposed and is termed as dipolar WP. However, it is important to note that while both higher‐order topological Weyl semimetals and the TWD phases exhibit hinge states, most of the higher‐order topological Weyl semimetals lack nontrivial WDs, and thus can not be classified as TWD phases.^[^ ^27^, ^29^, ^30^, ^49^ ^]^ Despite the realization of various higher‐order topological Weyl phases in the artificial crystals and phonon spectrum,^[^ ^28^, ^29^, ^30^, ^34^ ^]^ a TWD phase protected by a quantized quadrupole moment has yet to be reported in a crystalline material.

Drawing an analogy with electronic systems, the concept of topology has been applied to bosonic particles.^[^ ^50^, ^51^, ^52^, ^53^, ^54^ ^]^ Thus far, various topological phonon band crossings have been proposed, including phonons with nodal points^[^ ^6^, ^7^, ^8^, ^9^, ^10^, ^11^, ^12^, ^13^, ^14^ ^]^ and high‐dimensional geometric shapes.^[^ ^15^, ^16^, ^17^, ^18^, ^19^, ^20^ ^]^ Over 10 000 crystalline materials have been predicted to be topological phononic materials from the theory,^[^ ^20^ ^]^ and some of them have been verified from the experiment.^[^ ^14^, ^15^, ^16^, ^17^ ^]^ It is feasible to integrate higher‐order topology into topological phonons, such as Weyl phonons, and the exploration of higher‐order topological phonons, specifically higher‐order Weyl phonons, has emerged as a prominent focus in condensed‐matter research.^[^ ^27^, ^49^, ^55^, ^56^, ^57^, ^58^ ^]^

In this work, we focus on the TWD phase in the phonon spectrum of the crystalline materials and discover a nontrivial unconventional WD phase in Y(OH)_3_ with a space group (SG) *P*6_3_ (No. 173). The nontrivial unconventional WD phase is distinguished from all previously reported higher‐order Weyl phases.^[^ ^27^, ^28^, ^29^, ^30^, ^34^, ^37^ ^]^ The two WDs in Y(OH)_3_ are connected by time‐reversal symmetry (T), and each WD is formed by a charge‐3 WP with charge of –3 on the Γ‐*A* path, along with three charge‐1 WPs with charge of +1 on the three *M*‐*L* paths. The *k*_*z*_ plane, which is present both inside and outside of the two WDs, exhibits a nontrivial quantized quadrupole moment of 1/2 (*q*_*xy*_ = 1/2) and a trivial quadrupole moment of 0 (*q*_*xy*_ = 0), respectively. This directly shows that the WD in Y(OH)_3_ is a nontrivial *Z*_2_ WD. Due to the presence of both first‐ and second‐order topological charges, the boundary states in crystal Y(OH)_3_ are also quite unique. Y(OH)_3_ features 2D sextuple‐helicoid Fermi‐arc states on the top and bottom surfaces as well as 1D hinge states that connect the two TWDs along the side hinges.

## 2 Results and Discussion

### 2.1 Unconventional WP and WD Phonon in Y(OH)_3_

**Figure 2:**
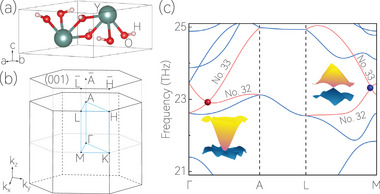
a) Crystal structure of Y(OH)_3_. b) Bulk BZ and (001) surface BZ of Y(OH)_3_. c) Enlarged phonon dispersion curve of Y(OH)_3_ in the frequency range of 21‐25 THz. In (c), the red and blue balls represent the charge‐3 WP and charge‐1 WP, respectively. The 3D plots of the phonon dispersion curve around the two doubly degenerate crossing points are also exhibited.

The crystal structure of crystalline material Y(OH)_3_, which belongs to SG No. 173, is of the hexagonal‐type, as shown in **Figure** **2**a. The operators in SG No. 173 are generated by a sixfold screw rotation $\left\{C_{6z}|00\frac{1}{2}\right\}$, which results in the emergence of *C*_3*z*_ and $\left\{C_{2z}|00\frac{1}{2}\right\}$. Moreover, the Y(OH)_3_ has T. The optimized lattice constants of Y(OH)_3_ are *a* = *b* = 6.19 Å and *c* = 3.50 Å. The Wyckoff positions of Y, O, and H atoms are 2*b*, 6*c*, and 6*c*, respectively. The bulk Brillouin zone (BZ) and (001) surface BZ of Y(OH)_3_ are illustrated in Figure 2b. The Y(OH)_3_ is dynamically stable, although its phonon spectrum exhibits small imaginary frequencies of approximately ‐0.1 THz around the Γ point,^[^ ^59^ ^]^ as shown in Figure S1 (Supporting Information). Information regarding the computational methods is also available in Supporting Information.

**Figure 3:**
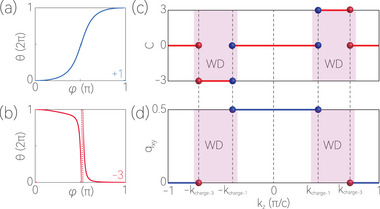
Panels (a) and (b) evolutions of the average position of the Wannier center for charge‐1 and charge‐3 WPs. Panels (c) and (d) evolutions of the Chern number C and quadrupole moment *q*_*xy*_ for the gaps between Nos. 32 and 33 phonon bands along the *k*_*z*_.

Here, we concentrated on the phonon bands within the frequency range from 21 to 25 THz, as illustrated in Figure 2c. Within this range, we observed two doubly degenerate WPs located on the Γ‐*A* and *L*‐*M* paths, formed by 32nd and 33rd bands, which are represented by red and blue balls, respectively. A careful scan reveals that apart from these two kinds of WPs, the 32nd and 33rd bands did not form any other band degeneracies. Thus, there are only eight WPs within the target frequency range. Then, according to the Nielsen–Ninomiya no‐go theory^[^ ^60^, ^61^ ^]^ and the encyclopedia of emergent particles,^[^ ^5^ ^]^ we can conculde that the WPs on the Γ‐*A* path are unconventional charge‐3 WPs with the same topological charge, as the WPs on the *L*‐*M* path must be charge‐1 WPs.^[^ ^5^ ^]^ This is confirmed by the Wilson‐loop calculations, which is performed on the spheres enclosing the WPs. As shown in **Figure** **3**a,b, the WP on the Γ‐*A* path possesses a topological charge of –3, whereas the WPs on the *L*‐*M* path have charge of +1. Moreover, we find that the *k*_*z*_‐positions of the charge‐3 WPs and charge‐1 WPs are located at *k*_charge‐3 WP_ ≃ ±0.272π/c and *k*_charge‐1 WP_ ≃ ±0.08π/c, respectively (see the Supporting Information for further details). Note that the two charge‐3 WPs are connected by T, and the six charge‐1 WPs are connected by the *C*_3*z*_ and T, as illustrated in Figure 1d.

A 3D system can be viewed as a collection of infinite 2D subsystems that depend on *k*_*z*_. Generally, one can calculate the Wilson loop and nested Wilson loop to identify the first‐order and second‐order topological properties of these 2D subsystems, respectively.^[^ ^27^, ^28^ ^]^ We first study the first‐order topology of the *k*_*z*_‐fixed 2D subsystems by calculating the Chern numbers (C). The C as a function of *k*_*z*_ is plotted in Figure 3c. One observes that in the regions where |*k*_*z*_| > |*k*_charge‐3 WP_| and |*k*_*z*_| < |*k*_charge‐1 WP_|, the 2D planes have C=0. However, in the region where |*k*_charge‐1 WP_| < |*k*_*z*_| < |*k*_charge‐3 WP_|, the planes have C=±3.

For the 2D subsystems with C=0, they have trivial first‐order topological properties, but they may possess nontrivial higher‐order topological properties. Particularly, the combination of one charge‐3 WP and three charge‐1 WPs above (below) *k*_*z*_ = 0 plane can be considered as an unconventional WD. Since the topological charge of the WD is zero, the WD has a trivial first‐order topology. However, due to the presence of *C*_2*z*_, each *k*_*z*_ plane is featured with a second‐order topological charge known as quantized quadrupole moment *q*_*xy*_(*k*_*z*_). *q*_*xy*_(*k*_*z*_) has two topologically inequivalent values: 0 and 1/2. *q*_*xy*_ = 0 and *q*_*xy*_ = 1/2 correspond to a normal insulator and a HOQTI, respectively. Therefore, the topological character of the WD can be characterized by the difference of the quantized quadrupole moment on two *k*_*z*_ planes that enclose the WD,

(1) $$Q_{WD}=q_{xy}\left(k_{z}^{>}\right)-q_{xy}\left(k_{z}^{<}\right)\;\;\;\mathrm{mod}\;\;\;1,$$

where $|k_{z}^{>}|\in \left(0.272\pi /c,\pi /c\right)$, $|k_{z}^{<}|\in \left(0,0.08\pi /c\right)$, and *q*_*xy*_(*k*_*z*_) is encoded in the determinant of the nested Wilson loop operator obtained in each *k*_*z*_ plane.^[^ ^46^ ^]^

As shown in Figure 3d, one finds that the planes in the region where |*k*_*z*_| < |*k*_charge‐1 WP_| host a nontrivial *q*_*xy*_ = 1/2, whereas the planes in the region where |*k*_*z*_| > |*k*_charge‐3 WP_| host a trivial *q*_*xy*_ = 0. Therefore, the topological charges of the two unconventional WDs are obtained as *Q*_*WD*_ = 1/2, showing the two unconventional WDs are topologically nontrivial. This also implies that the WPs in Y(OH)_3_ are more robust than those in conventional Weyl semimetals. It is well known that two WPs with opposite topological charges will annihilate when they come together. However, in stark contrast, even if the charge‐3 WP and the three charge‐1 WPs above (below) *k*_*z*_ = 0 plane meet together, they will not annihilate to form an insulator phase, as they remain nontrivial as a whole and are topologically protected by the quantized quadrupole moment.

Hence, the Y(OH)_3_ exhibits a variety of topological phases. The planes in the region where |*k*_*z*_| > |*k*_charge‐3 WP_|, with C = 0 and *q*_*xy*_ = 0, are NIs. The planes in the region where |*k*_charge‐1 WP_| < |*k*_*z*_| < |*k*_charge‐3 WP_|, with C = ±3, are QAHIs,^[^ ^62^, ^63^, ^64^ ^]^ which belong to first‐order 2D topological insulators. The planes in the region where |*k*_*z*_| < |*k*_charge‐1 WP_|, with C = 0 and *q*_*xy*_ = 1/2, are HOQTIs,^[^ ^46^, ^65^ ^]^ which belong to second‐order 2D topological insulators. At the boundaries of these insulator phases are the charge‐3 and charge‐1 WPs, which give rise to unconventional WDs.

### 2.2 Unconventional Boundary States in Y(OH)_3_

**Figure 4:**
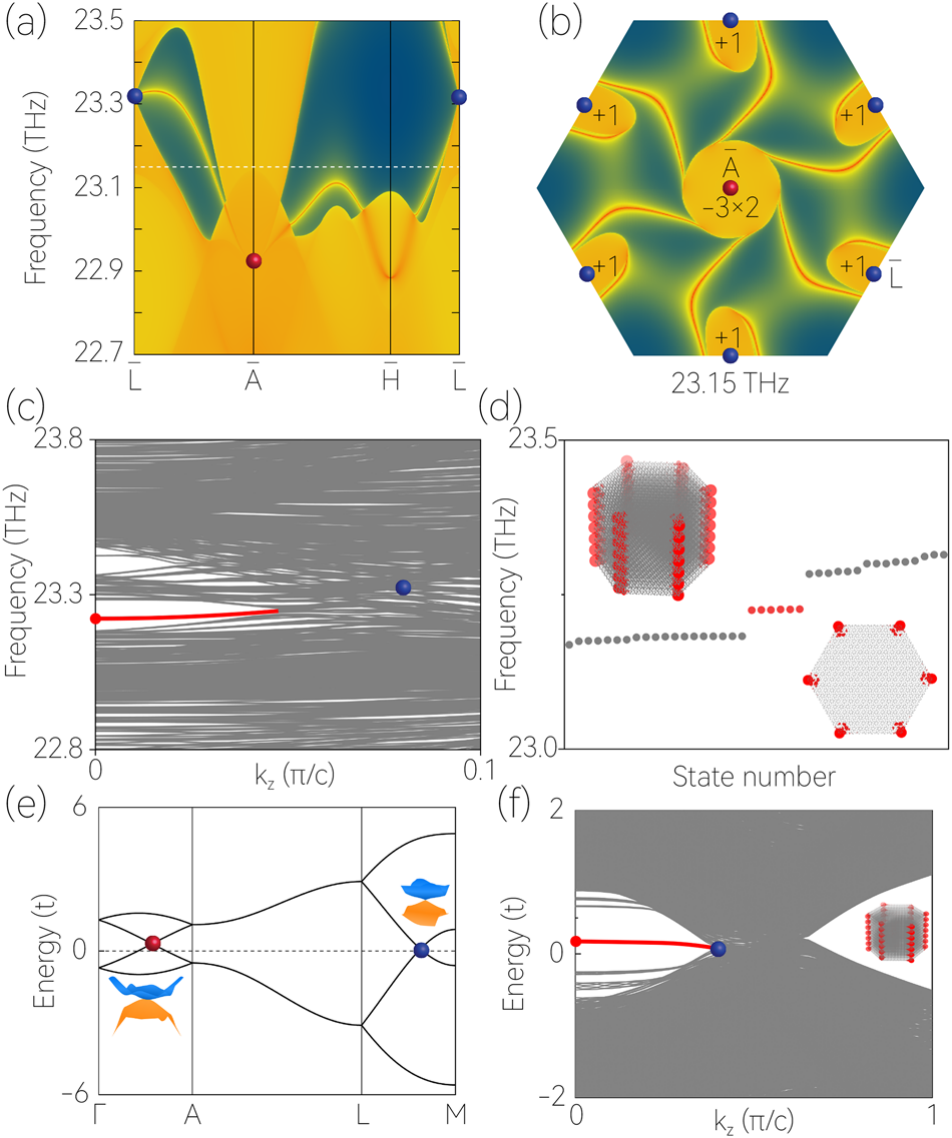
a) Projected spectrum of Y(OH)_3_ on the (001) surface. b) Constant‐frequency slice at 23.15 THz. c) Enlarged phonon spectrum (in the *k*_*z*_ direction) within the frequency range from 22.8 to 23.8 THz for a sample of Y(OH)_3_ with a tube‐like geometry. The hinge states are highlighted by the red curves. d) Frequency spectrum for a sixfold degenerate state (red dot) at *k*_*z*_ = 0. These insets are the spatial distributions for the sixfold degenerate state. e) Bulk band structure of the tight‐binding model. These insets are 3D plots of bands around the charge‐3 WP and charge‐1 WP. f) The spectrum of a 1D tube geometry (in the *k*_*z*_ direction). The inset is the spatial distribution for the state at the *k*_*z*_ = 0.

The coexistence of the first‐order and second‐order topologies is manifested in the multi‐dimensional boundary modes. First, we calculated the surface spectrum of Y(OH)_3_, which is projected on the (001) plane along the $\bar{L}$‐$\bar{A}$‐$\bar{H}$‐$\bar{L}$ paths (see **Figure** **4**a). As shown in Figure 4b, the two charge‐3 WPs are projected to the same position, i.e., $\bar{A}$ surface point in the (001) surface BZ. Consequently, the two charge‐3 WPs necessitate the existence of six surface arcs connected to the surface $\bar{A}$ point, resulting in 2D sextuple‐helicoid arc‐shaped surface states.

Second, we established a sample of Y(OH)_3_ with a tube‐like geometry using the phononic tight‐binding model and calculated the phonon dispersion curve along the *k*_*z*_ direction, as shown in Figure 4c. Since the two nontrivial unconventional WDs have *Q*_*WD*_ = 1/2, they will feature a second‐order boundary state along the hinge of the Y(OH)_3_ with a tube‐like geometry. Indeed, a phononic hinge band, shown by a red line, can be seen in Figure 4c and connects the projections of nontrivial unconventional WDs. To further confirm it, we select a sixfold degenerate state at the *k*_*z*_ = 0 (red dot in Figure 4c) and plot the spatial distribution for the state in Figure 4d under different viewpoints. Evidently, the sixfold degenerate state is distributed at the six 1D hinges of the tube sample, demonstrating the existence of the second‐order boundary states.

## 3 Discussion and Conclusion

For a better understanding of the topological properties, we also develop a simple effective lattice model of the nontrivial unconventional WD phase. Based on the material Y(OH)_3_, we construct a four‐band tight‐binding model of SG No. 173, in which two charge‐3 WPs and six charge‐1 WPs appear on the Γ‐*A* path and *L*‐*M* path, as shown in Figure 4e. One charge‐3 WP and three charge‐1 WPs together form a nontrivial unconventional WD phase with *Q*_*WD*_ = 1/2, which leads to the appearance of the hinge states (see Figure 4f). The details of the Hamiltonian are given in Supporting Information.

In summary, with the help of first‐principles calculations and symmetry analysis, we propose a new higher‐order topological phonon state, which integrates an unconventional WD phonon with higher‐order topology. *P*6_3_ type Y(OH)_3_ has been selected as the first crystalline material to host the nontrivial unconventional WD phase in its optical phonon spectrum. Moreover, the two WDs in Y(OH)_3_ exhibit the sextuple‐helicoid surface and hinge boundary states from the nonzero topological charges (±6) and the nontrivial quadrupole moment (*Q*_*WD*_ = 1/2), respectively. Our findings reveal a new type of higher‐order topological phonons and demonstrate that the phonon spectra can serve as an excellent platform for investigating nontrivial unconventional WDs and multi‐dimensional boundary states.

## Conflict of Interest

The authors declare no conflict of interest.

## Supporting information

Supporting Information

## Data Availability

The data that support the findings of this study are available on request from the corresponding author. The data are not publicly available due to privacy or ethical restrictions.
