# Supplementary material for: Unconventional Topological Weyl Dipole Phonon
Source: Adv Sci (Weinh). 2025 Jun 17;12(32):e04812. doi: 10.1002/advs.202504812 (PMC12407364; doi:10.1002/advs.202504812)
Supplement: Supplementary file 1 — Supporting Information [file ADVS-12-e04812-s001.pdf]

ADVANCED SCIENCE
Open Access

Supporting Information

for *Adv. Sci.*, DOI 10.1002/advs.202504812

Unconventional Topological Weyl Dipole Phonon

Jianhua Wang, Yang Wang, Feng Zhou, Wenhong Wang, Zhenxiang Cheng, Shifeng Qian, Xiaotian Wang* and Zhi-Ming Yu*

Supporting Information for "Unconventional topological Weyl dipole phonon"

Jianhua Wang, Yang Wang, Feng Zhou, Wenhong Wang, Zhenxiang Cheng, Shifeng Qian, Xiaotian Wang*, Zhi-Ming Yu*

J. Wang, F. Zhou, W. Wang
Institute of Quantum Materials and Devices, Tiangong University, Tianjin 300387, China
J. Wang, Z. Cheng, X. Wang
Email:xiaotianw@uow.edu.au
Institute for Superconducting and Electronic Materials, Faculty of Engineering and Information Sciences, University of Wollongong, Wollongong 2500, Australia
Y. Wang, Z.-M. Yu
Key Lab of Advanced Optoelectronic Quantum Architecture and Measurement (MOE), Beijing Key Lab of Nanophotonics & Ultrafine Optoelectronic Systems, and School of Physics, Beijing Institute of Technology, Beijing 100081, China
S. Qian
Anhui Province Key Laboratory for Control and Applications of Optoelectronic Information Materials, Department of Physics, Anhui Normal University, Wuhu, Anhui 241000, China
Email:qiansf@ahnu.edu.cn

S1 Computational Methods

First-principles calculations were conducted using the Vienna *ab initio* simulation package (VASP) [1]. The Perdew-Burke-Ernzerhof (PBE) generalized gradient approximation (GGA) [2] was used to calculate the exchange energy. The projector-augmented wave pseudopotentials were used to simulate the interaction between ions and valence electrons. All calculations were performed with a plane-wave cutoff of 500 eV, and the convergence criterion for the electronic self-consistence loop was set to be 1×10^{-6} eV on the $7\times7\times10$ Monkhorst-Pack k-point mesh, and for the structural relaxation, the Hellmanne-Feynman forces on each atom were taken to be -0.01 eV/Å. The phonon dispersion calculations were fulfilled by the density functional perturbation theory [3,4] coded in the PHONOPY package. A $2\times2\times3$ supercell was adopted to calculate force constants. Furthermore, based on the first-principles calculation, the surface modes were obtained by constructing a phonon tight-binding model via WANNIERTOOLS [5].

A 3D system can be viewed as k_z-dependent 2D subsystems. One can calculate the Wilson loop and nested Wilson loop to identify the first-order and higher-order topological properties of the 2D subsystem, respectively. The Wilson loop operator of k_x has the form of

$$W_{1(k_x,k_y)\rightarrow(k_x+2\pi,k_y)} = \lim_{N\rightarrow\infty} F_0 F_1 \cdots F_{N-2} F_{N-1}, \tag{S1}$$

where F_i is the overlap matrix whose matrix elements is defined by

$$[F_i]_{mn} = \langle u_m\left(2\pi(i)/N, k_y\right) \mid u_n\left(2\pi(i+1)/N, k_y\right)\rangle . \tag{S2}$$

The topological property is encoded in the phase $\theta_m\left(k_y\right)$ of the eigenvalues of the Wilson loop operator. The spectrum of the $\theta - k$ diagram represents the evolution of the Wannier centers and the Chern number can be defined by the winding number of the Wannier centers.

The Chern number of 0 indicates that these two k_z-dependent 2D subsystems have trivial first-order topological properties, but they may possess nontrivial higher-order topological properties. To examine the higher-order topological properties, we calculated the nested Wilson loop to determine the quadrupole moment q_{xy}. When the Wilson loop operator is diagonalized as

$$W_{(k_x+2\pi,k_y)\leftarrow(k_x,k_y)} = \sum_i \left|\nu_i\left(k_y\right)\right\rangle e^{i\theta_i(k_y)} \left\langle\nu_i\left(k_y\right)\right| , \tag{S3}$$

using the set of eigenvectors corresponding to the sub-band of the Wilson loop operator at the $\theta = \pi$, the nested Wilson loop operator can be defined as

$$W_{2(k_y)\to(k_y+2\pi)} = \lim_{N\to\infty} \tilde{F}_0 \tilde{F}_2 \cdots \tilde{F}_{N-2} \tilde{F}_{N-1}, \tag{S4}$$

where

$$\left[\tilde{F}_i\right]_{mn} = \langle \nu_m(2\pi(i)/N) \mid \nu_n(2\pi(i+1)/N)\rangle \,. \tag{S5}$$

The quadrupole moment q_{xy} is encoded in the determinant of the nested Wilson loop operator. The symmetry operator C_{2z} enforced the quantized quadrupole moment q_{xy}. Therefore, the nested Wilson only has two values, 0 and π, where π corresponds to a HOQTI, which hosts a non-zero quantized quadrupole moment $q_{xy} = 1/2$.

S2 Tight-binding model

Here, we construct a tight-binding model for space group 173 with the p_x and p_y orbitals. The tight-binding Hamiltonian is given by

$$\begin{aligned}
\mathcal{H}_{(\vec{k})} = {} & (r_3 + r_6)\chi_1(\vec{k})\Gamma_{0,0} - \frac{1}{2}(r_3 - r_6)\chi_1(\vec{k})\Gamma_{0,3} - \frac{\sqrt{3}}{2}(r_3 - r_6)(\cos k_x - \cos k_y)\Gamma_{0,1} \\
& - t_1(-2 + \chi_1(\vec{k}))\cos k_z \Gamma_{1,0} + 4t_1 \cos\frac{k_z}{2}\chi_2(\vec{k})\Gamma_{2,1} + (2t_1 + r_6\chi_1(\vec{k}))\sin\frac{k_z}{2}\Gamma_{1,2} \\
& - 4r_6 \sin\frac{k_z}{2}\chi_2(\vec{k})\Gamma_{2,2} + \frac{1}{2}[r_6(\cos k_x + \chi_1(\vec{k})) + \frac{\sqrt{3}}{2}t_1(\cos k_x + 2\cos(k_x + k_y))]\cos\frac{k_z}{2}\Gamma_{1,1} \\
& - \frac{1}{2}(t_1(\chi_1(\vec{k}) - 3\cos k_y) + \sqrt{3}r_6(-\cos k_y + \cos(k_x + k_y)))\cos\frac{k_z}{2}\Gamma_{1,3} \\
& + \frac{1}{2}[r_6\chi_3(\vec{k}) - \sqrt{3}t_1(\sin k_x + \sin(k_x + k_y))]\cos\frac{k_z}{2}\Gamma_{2,1} \\
& + \frac{1}{2}[t_1(\chi_3(\vec{k}) - 3\sin(k_x + k_y)) + \sqrt{3}r_6(\sin k_y + \sin(k_x + k_y))]\cos\frac{k_z}{2}\Gamma_{2,3},
\end{aligned} \tag{S6}$$

where $r_3 = 0.5$, $r_6 = -0.4$, $t_1 = 1$,

$$\chi_1(\vec{k}) = \cos k_x + \cos k_y + \cos(k_x + k_y), \tag{S7}$$

$$\chi_2(\vec{k}) = \sin\frac{k_x}{2}\sin\frac{k_y}{2}\sin\frac{k_x + k_y}{2}, \tag{S8}$$

$$\chi_2(\vec{k}) = \sin k_x - 2\sin k_y + 2\sin(k_x + k_y), \tag{S9}$$

$$\Gamma_{i,j} = \sigma_i \otimes \sigma_j. \tag{S10}$$

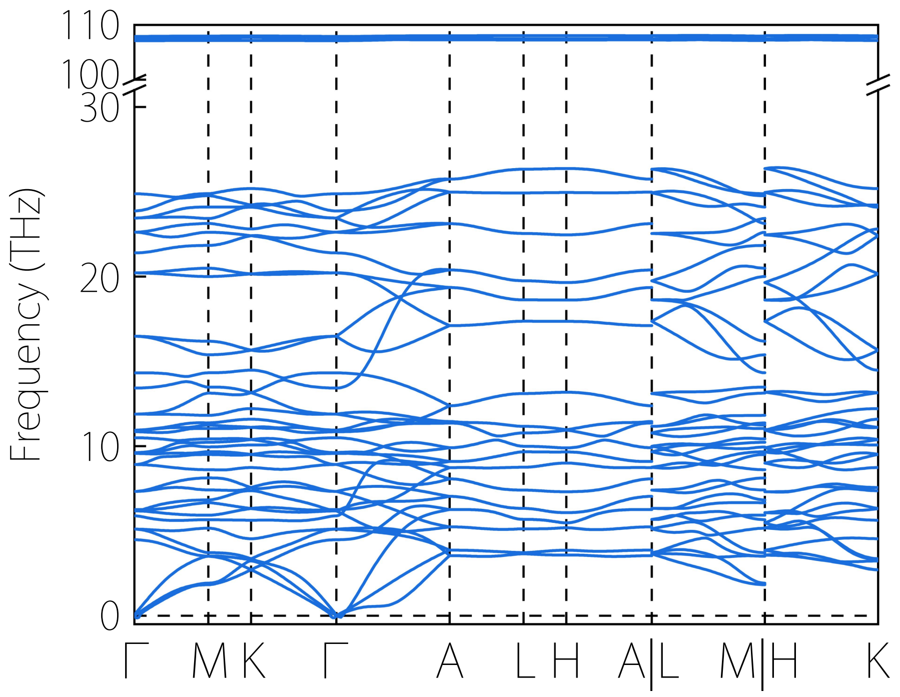

Figure S1: Phonon dispersion curve of $Y(OH)_3$.

Table S1: The momentum positions and topological charges of all WPs formed by the 32nd and 33rd.

No.	Momentum position	Topological charge
1	(0.5, 0, 0.04)	+1
2	(0.5, 0, -0.04)	+1
3	(0.5, -0.5, 0.04)	+1
4	(0.5, -0.5, -0.04)	+1
5	(0, 0.5, 0.04)	+1
6	(0, 0.5, -0.04)	+1
7	(0, 0, 0.136)	-3
8	(0, 0, -0.136)	-3

References

[1] J. Hafner, *J. Comput. Chem.* **2008**, 29, 2044.

[2] J. P. Perdew, K. Burke, M. Ernzerhof, *Phys. Rev. Lett.* **1996**, 77, 3865.

[3] S. Baroni, P. Giannozzi, A. Testa, *Phys. Rev. Lett.* **1987**, 58, 1861.

[4] X. Gonze, C. Lee, *Phys. Rev. B* **1997**, 55, 10355.

[5] Q. Wu, S. Zhang, H.-F. Song, M. Troyer, A. A. Soluyanov, *Phys. Chem. Comm.* **2018**, 224, 405.
